# Colorectal Cancer, Liver Metastases and Biotherapies

**DOI:** 10.3390/biomedicines9080894

**Published:** 2021-07-26

**Authors:** Daniel-Clement Osei-Bordom, Sivesh Kamarajah, Niki Christou

**Affiliations:** 1 Department of General Surgery, Queen Elizabeth Hospital, University Hospitals Birmingham, Birmingham B15 2TH, UK; danieloseibordom@gmail.com (D.-C.O.-B.); sivesh.kamarajah@uhb.nhs.uk (S.K.); 2 Institute of Immunology and Immunotherapy, University of Birmingham, Birmingham B15 2TT, UK; 3 NIHR Birmingham Biomedical Research Centre, Centre for Liver and Gastroenterology Research, University of Birmingham, Birmingham B15 2TT, UK; 4 Department of General Surgery, University Hospital of Limoges, 87000 Limoges, France; 5 EA3842 CAPTuR Laboratory “Cell Activation Control, Tumor Progression and Therapeutic Resistance”, Faculty of Medicine, 2 Rue du Docteur Marcland, 87025 Limoges, France

**Keywords:** colorectal cancer, liver metastases, immunotherapy, chemotherapy and biological agents

## Abstract

(1) Background: colorectal cancer (CRC) is one of the deadliest causes of death by cancer worldwide. Its first main metastatic diffusion spreads to the liver. Different mechanisms such as the epithelial–mesenchymal transition and angiogenesis are the characteristics of this invasion. At this stage, different options are possible and still in debate, especially regarding the use of targeted therapeutics and biotherapies. (2) Methods: A review of the literature has been done focusing on the clinical management of liver metastasis of colorectal cancer and the contribution of biotherapies in this field. (3) Results: In a clinical setting, surgeons and oncologists consider liver metastasis in CRC into two groups to launch adapted therapeutics: resectable and non-resectable. Around these two entities, the combination of targeted therapies and biotherapies are of high interest and are currently tested to know in which molecular and clinical conditions they have to be applied to impact positively both on survival and quality of life of patients.

## 1. Introduction

Colorectal cancer (CRC) is the third most common cancer type, only behind breast cancer and lung cancer in females and prostate cancer and lung cancer in males. CRC is also a leading cause of cancer-related deaths globally. The liver is of anatomical and physiological importance in regard to the natural course of CRC. The liver and lung are the commonest sites for CRC metastasis to occur. The liver’s predominant blood supply arises from the confluence of the GI tract supplying blood vessels via the hepatic portal vein; this circulation aids the transference of the colorectal cancer cells to the hepatic parenchyma, migrating and forming colorectal metastases in the liver. The overall prognosis and survival of patients with CRLM are poor, and most patients initially are unable to undergo surgery.

The management of colorectal metastases of the liver varies on CRC disease burden, patient suitability, clinical correlation and appropriateness of treatments decided by cancer multidisciplinary team (MDT). Conventional treatment methods of CRRLM include hepatic resection with or without chemo-radiotherapy (neoadjuvant or adjuvant) and other conservative management. The development of novel and effective biotherapies in conjunction with chemotherapies for colorectal metastases have changed the natural course of CRC by enhancing the host’s own immunological anti-tumour responses against CRC and already shown to have a good potential and improve the prognosis of the disease. Treatment strategies utilizing and evaluating the benefit of the use of biotherapies in CRLM of the liver remain unclear. In this review, we will examine what biotherapies are available, and the benefits of implementing biotherapies in the context of CRLM. This review will highlight the current strategies and available biotherapies in use or in trials for the management and treatment of CRC metastases of the liver.

## 2. Biology of the Metastatic Colorectal Cancer

Colorectal adenocarcinoma forms the largest disease type for CRCs. These adenocarcinomas derive from sequential changes involving mutations and key oncogenes and the loss of suppressor genes driving changes of adenomas into adenocarcinomas [1]. These mutational changes include KRAS and inactivation of tumour suppressor gene p53, which drive the pathogenesis of adenocarcinoma from adenomas [2]. The adenoma–adenocarcinoma sequence highlights significant avenues for targeted biological therapeutics. KRAS primarily belongs to the family of GTPases. Upon activation, KRAS induces the mitogen-activated protein kinase (MAPK) cascades and facilitates the transmission of signals from the cell membrane to the nucleus; the RAS gene, through downstream signalling, activates RAF proteins (including ARAF, BRAF, and RAF-1) [3,4].

The signalling pathway of the RAS–RAF–MEK–MAPK cascade regulates gene transcription controlling cancer cell proliferation, survival, migration and angiogenesis, abetting the progression of colorectal cancer (CRC) and promotes metastasis. Therefore, direct inhibition of MEK binding and effector function, consequently, may uncover a promising targeted therapeutic strategy for CRCs. There are several targeted inhibitors that are currently under evaluation in clinical trials showing initial clinical activity in a variety of tumours, including mCRC [4,5,6]. Specific markers that targeted therapies are aimed against include: Epidermal Growth Factor Receptor (EGFR), BRAF and tyrosine kinases, amongst others.

Resistance to molecular biological targeted therapies can be detected in the early and advanced stages of the disease, and this can occur in an estimated prevalence of up to 45% in patients with CRLM. Studies have shown that such mutations are independently associated with worse survival and poor overall outcomes [7,8].

**Figure 1:**
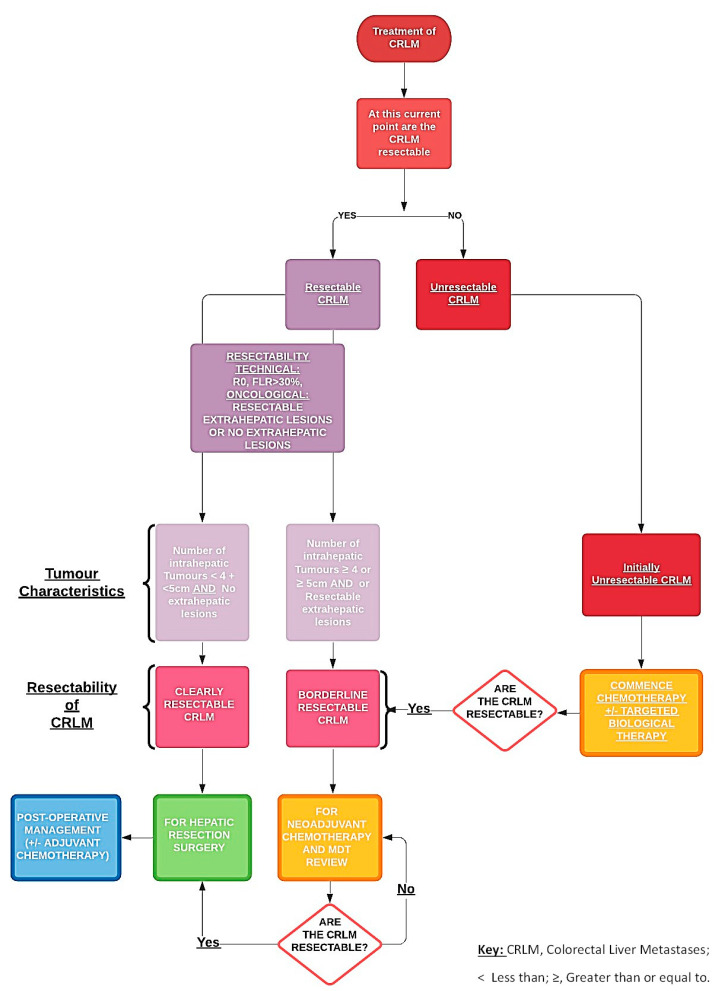
Pathway of managing resectable and unresectable colorectal liver metastases.

The use of biological therapies in combination with chemotherapy is an essential option towards “conversional” strategies that aim to transform initially unresectable CRLM to resectable CRLM, and thereby has evidently increased the proportion of patients eligible for hepatic resection. Along with the advancements in the amalgamation of perioperative and surgical management, the use of effective chemotherapies, targeted biological therapies and novel methods of delivering these targeted therapies locally (e.g., hepatic intra-arterial chemotherapy, RFA, stereotactic radiotherapy) [9], as we strive to decrease perioperative morbidity and mortality, increase long-term survival by increasing the number of patients who are able to undergo complete hepatic resections (Figure 1).

Chemotherapy is a chemically constituted anticancer therapy that acts by reducing the tumour burden and facilitates the destruction of rapidly growing cancer cells in the body. In colorectal cancer, the most used are shown in Table 1.

**Figure 2:**
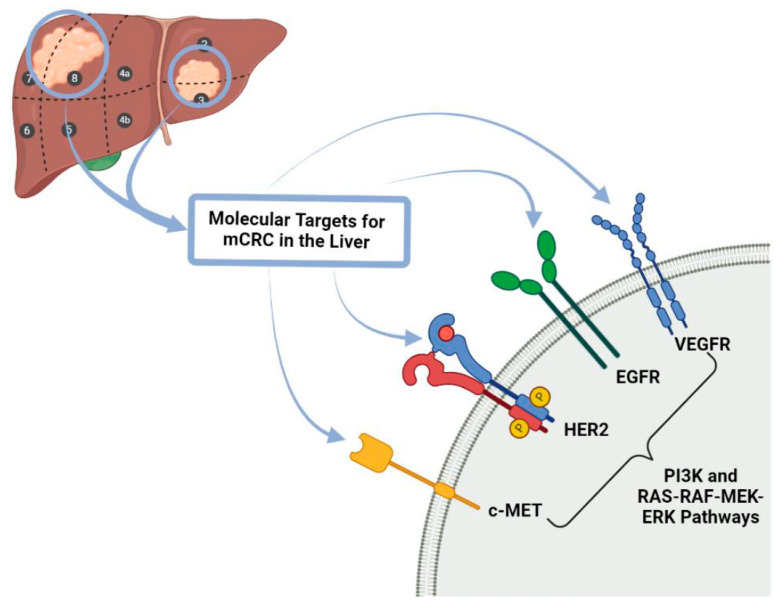
Molecular Targets for mCRC in the liver. c-MET: tyrosine-protein kinase Met receptor, HER 2: Human Epidermal Growth Factor Receptor-2, EGFR: Epidermal growth factor receptor, VEGFR: Vascular Endothelial Growth Factor Receptor PI3K: Phosphoinositide 3-kinase, RAS-RAF-MEK-ERK pathways also known MAPK/ERK pathway corresponds to the Ras/Raf/Mitogen-activated protein kinase/ERK kinase (MEK)/extracellular-signal-regulated kinase (ERK) cascade couples signals from cell surface receptors to transcription factors, regulating gene expression and mCRC: metastatic colorectal cancer.

With regards to targeted therapies used in the treatment of colorectal cancer and their metastases, the main forms of this type of therapy are Tyrosine Kinase Inhibitors, BRAF (proto-oncogene B-Raf or v-Raf murine sarcoma viral oncogene homolog B) inhibitors and epidermal growth factor receptor (EGFR) inhibitors (Figure 2).

Tyrosine Kinase Inhibitors, also known as kinase inhibitors, located on cellular surfaces and intracellularly, are proteins involved in cell to cell signalling and are key elements in cell function. Kinase inhibitors like Regorafenib block several kinase proteins that assist the tumour cell growth and tumour blood vessel development, similar to anti-angiogenic inhibitors (Table 2). Blocking these proteins can impair tumour growth and function. *BRAF* gene and its proteins play a fundamental role in colorectal cancer (CRC). Some CRC cells produce abnormal BRAF proteins that promote tumour growth, hence being an important therapeutic target. A limiting factor in using BRAF inhibitors is that BRAF inhibitors are unlikely to work on patients with colorectal cancers that have normal BRAF genes. Epidermal growth factor receptor (EGFR) is a protein-based receptor that also assists cancer cell growth. Primarily, EGFR inhibitors are used in patients with advanced colon or rectal cancers.

Biotherapies are a form of therapies encompassed under the umbrella term of immunotherapies in which these agents utilize and enhance a patient’s own immune system to treat cancer.

## 3. Current Management of Liver Metastases from Colorectal Cancers

### 3.1. Initial Assessment

Patients with suspected CRC will have undergone several pre-operative evaluations, including have CT Thorax, Abdomen and Pelvis with Contrast. Occasionally, these preliminary imaging modalities identify the presence of a potential primary CRC and/or lesions that are detected within the liver (metachronous or synchronous) [10,11]. We then opt for additional imaging in the format of a contrast magnetic resonance imaging (MRI) of the Pelvis (in case of rectal cancers) and an MRI of the Liver with contrast. MDT involving colorectal surgeons, HPB surgeons, radiologists and oncologists decide on the most favourable management plan given the patient’s pathology encompassing CRC resection, chemotherapy with or without biological agents and hepatic resection. Resectable hepatic disease is considered for pre-operative chemotherapy followed by resection or attempt for immediate curative resection (R0) if pathology meets resectability criteria [12]: (i) CRLM must be resectable with negative margins and allow for the preservation of at least two contiguous liver segments with intact inflow, venous outflow and biliary drainage; (ii) the volume of this future liver remnant (FLR) depends on the functionality (~30% of total liver volume for a normal liver, FLR > 30%, if liver fibrosis is present). The tumour number and location and position are determined with imaging pre-operatively and intra-operatively as previously discussed, and for deeper-seated lesions in the liver parenchyma and smaller than 2 cm in diameter, combined radiofrequency ablation (RFA) is frequently used to minimize liver tissue loss and limits post-resection hepatic dysfunction.

Hepatic resection is considered the primary operation for mCRC if patients have a high tumour burden or with primary tumours located in the rectum, which will require pre-operative chemo-radiotherapy [13,14]. Major hepatic resection is defined as the resection of 3 or more hepatic segments associated with an increased mortality rate of ~15%. The use of neoadjuvant chemotherapy in combination with targeted biotherapies has shown to increase resectability in complex CRLM and unresectable CRLM prior to hepatic resection. In spite of the neoadjuvant therapies, there a credible risk associated with chemotherapy-associated liver injury (CALI), which can impact patient suitability for hepatic resection [15,16]. Hepatic changes such as sinusoidal obstruction, peri-portal inflammation, and steatohepatitis are associated with neoadjuvant therapies, which can alter a patient’s overall outcome [17]. Specifically, the direct use of oxaliplatin is linked to the development of sinusoidal obstruction syndrome in up to 38% of patients, while steatosis and steatohepatitis complicated 9.3% of patients receiving irinotecan. Patients with the following complications were more likely to suffer post-operative complications (severe sinusoidal dilation (OR 1.73) or steatohepatitis (OR 2.08)) [12].

### 3.2. Perioperative Chemotherapy

The use of chemotherapy in patients with CRLM is usually reserved for patients with borderline resectable disease and patients with unresectable disease (Figure 1). Chemotherapy aids in improving overall patient survival outcomes by facilitating hepatic resections. Recommended guidelines suggest that using FOLFOX or CAPOX, essentially oxaliplatin-based chemotherapy during the neoadjuvant period, is the ideal choice for patients with borderline resectable CRLMs, while FOLFIRI or FOLFOXIRI are alternative options (Table 1) [18]. During the period of delivery of chemotherapy, rigorous interval imaging is required to elicit the timepoint at which the CRLM become clearly resectable from borderline resectable and prepare for hepatic resection [19]. Monitoring CRLM during the active treatment with chemotherapy with or without other targeted therapies is extremely important as there is a genuine risk of in this group of patients developing “disappearing CRLM”, which can complicate the treatment pathway of affected patients [20].

For unresectable disease, conversional chemotherapy with and without biological therapies is used to convert unresectable disease into resectable disease [21,22]. In standard systemic chemotherapy regimens with oxaliplatin +/− irinotecan-based regimens in combination with 5-FU (FOLFOX, FOLFIRI and FOLFOXIRI), studies have shown that chemotherapy has been able to facilitate resections in ~40% of initially unresectable patients [23,24,25]. Neoadjuvant chemotherapy in unresectable disease has demonstrated a reduction in the size of the liver metastases by ~50% in tumour mass: this was seen in upwards of 60% of patients with unresectable CLM with complete resection in 40% of these patients [19,26,27].

A recent systematic review and meta-analysis reviewed the effectiveness of using neoadjuvant chemotherapy plus molecular targeted therapy in unresectable CRLM [28]. The study identified that using chemotherapy plus targeted biological therapy for unresectable CRLM patients had an impact on the overall response rate (ORR). ORR is a measure of the proportion of patients whose disease reduced (partial response–PR) and/or disappears (complete response–CR) after treatment [29]. The study highlighted that patients who received chemotherapy plus molecular targeted therapy had a higher overall response rate when compared with patients using chemotherapy alone (68% vs. 43%), but evidence to suggest improved overall survival (OS) remains inconclusive [28,30].

The EPOC trial also evaluated the Progression-Free Survival (PFS) in patients with unresectable CRLM, which is an important measure in treated metastatic disease. PFS is used as a primary endpoint in trials evaluating the treatment of metastatic cancer. PFS is the length of time during and after the treatment (chemotherapy +/− targeted biological therapies) of unresectable CRLM that a patient lives with stable or improved metastatic disease. PFS for unresectable CRLM patients undertaking perioperative systemic chemotherapy plus EGFR-inhibitor (Cetuximab) vs. chemotherapy indicated that this cohort of patients, who received specifically chemotherapy plus cetuximab, essentially experienced worse PFS when compared with the chemotherapy control group (14.1 vs. 20.5 months in control) [12,22]. The trial concluded that cetuximab should not be given with perioperative chemotherapy regimens; however, the trial emphasized the use of other groups of targeted biological therapies that showed improved PFS and OS. Further combinations of systemic chemotherapeutics and targeted biotherapies were reviewed; Bevacizumab, a vascular endothelial growth factor (VEGF) inhibitor, was combined with FOLFIRI (Folinic acid, fluorouracil and irinotecan) as joint neoadjuvant therapy and yielded a response rate of 66.7% in resectable CRLMs; however, the survival outcomes and benefits are still to be determined.

The PERIMAX trial evaluated the benefits and limitations of a highly active chemotherapy +/− targeted biological therapy regimens in the perioperative and post-operative setting for resectable and unresectable CRC. Patients with resectable liver metastases were randomized into perioperative treatment with FOLFOXIRI and bevacizumab or post-operative FOLFOX, and patients with unresectable mCRC being randomized between FOLFOX and bevacizumab with or without irinotecan. Analysis suggests that the use of cetuximab plus chemotherapy had no impact on overall survival compared with chemotherapy alone for the unresectable CRLM group patients and in resectable CRLM patient cohort, cetuximab use adversely affected OS (HR = 0.95 and 2.35, respectively) [22]. Perioperative chemotherapy offers an opportunity to reduce cancer recurrence post-resection in approximately 70% of patients after resection and drives complete eradication of CRC, and thereby imparting a survival benefit [31,32].

### 3.3. Synchronous Disease

The liver along with the lung are the most common sites of the metastases of colorectal cancer. Studies have shown about 40–50% of CRC patients will develop liver metastases at a point during the course of CRC disease [33,34]. About 20% of patients often have established synchronous liver metastases when the diagnosis of primary colorectal cancer is made. Synchronous liver metastases are associated with poor outcomes likely caused by poor tumour biology and complex treatment plans [13,35]. Hepatic resection is a definitive treatment option to achieve long-term survival and overall survival, as studies have shown that after primary and secondary resections. CRLM resection offered an overall median survival of 3.6 years; 5- and 10-year survival ranged from 16% to 74% (median 38%) and 9% to 69% (median 26%), respectively. Hepatic resections are divided into selective staged resection, delayed resection or simultaneous resections, with a higher proportion of teams opting for selective staged resection of CRCs, as this is associated with fewer risks accompanied with operating at two sites in simultaneous resection [36,37]. Risks associated with simultaneous resection include intraperitoneal infection, anastomotic fistula and hepatic insufficiency, as well as associated higher mortality. Hepatic resections are divided into three groups: (1) sequential resection (SeR), in which surgical teams are able to resect colorectal cancer and liver metastases without delivering interval chemotherapy; (2) delayed resection (DeR) in which we deliver interval chemotherapy between staged colonic cancer resection and hepatic resection of CRC metastases; and (3) simultaneous resection (SiR), single-stage resection of primary colorectal cancer and liver metastases simultaneously. SiR is performed in patients who fulfil certain criteria (1) the primary tumour was located in the right colon regardless of the tumour disease burden of liver metastases; (2) the tumour disease burden was not heavy, and the tumour number was less than two if the primary tumour was located in the left colon or rectum [38,39].

A staged resection is an important option in managing patients with high tumour burden (tumour number greater than three), rectal tumours requiring chemo-radiotherapy, and patients with significant co-morbidities who all are unlikely to tolerate simultaneous resections [40,41]. DeR selects patients post-primary surgery with resectable liver metastases who were treated with chemotherapy prior to the second resection operation and patients with unresectable liver metastases who were treated with chemotherapy +/− targeted biological therapies and then further evaluated and discussed in MDT after interval [12,42].

### 3.4. The Issue of Disappearing Liver Metastases

Pre-operative chemotherapy in patients with resectable CRLM can drive the phenomenon of “disappearing” CRLM (DLM) in a quarter of resectable CRLM lesions [20]. This phenomenon is identified using imaging. Despite the positive radiological response, 80% of these lesions remain viable metastases [43]. The risk of DLM is a valid concern when commencing resectable patients on chemotherapeutic agents. CRLMs, particularly in resectable patients, ideally will need to be identified on pre-operative contrast-enhanced imaging, and initially, resection or ablative therapies should be considered as initial treatment. However, the majority of these DLMs are still apparent and identifiable intraoperatively under direct vision and use of intraoperative ultrasonography (IOUS) imaging [44,45]. In patients with high-risk lesions (e.g., deep parenchymal lesions and smaller lesions), often, MDTs opt to limit the cycles of chemotherapeutics and proceed to surgery first. In the event where we are unable to excise all the DLMs or other sites of metastatic disease, these patients often rigorously followed up and managed upon macroscopic recurrence with a staged resection approach [46].

### 3.5. Patients with Initially Resectable Disease

The use of chemotherapy in patients with initially resectable disease varies from one centre to another; there is still no strict guidelines in the approach to integrating hepatic mCRC resection with systemic chemotherapeutics [47,48]. If patients have four or fewer mCRC resectable lesions with a primary colorectal tumour, then often centres opt for resection first over chemotherapy, unless there is a predicted strong response against chemotherapy. Initial chemotherapeutic treatment is reserved for patients with good exercise tolerance, few co-morbidities, multi-lobar tumour involvement and regional lymph node involvement. These patients are then re-evaluated with interval imaging 6–8 weeks to assess response to the chemotherapy and then re-discussed in MDT prior to surgical resection.

Beppu et al. demonstrated in the EPOC trial highlighted that the optimal neoadjuvant combination regime for patients with initially resectable CRLM with RAS mutations as such FOLFOX, FOLFIRI and/or CAPOX with or without bevacizumab; FOLFIRI with or without cetuximab or panitumumab; or FOLFOX with or without panitumumab or cetuximab (if RAS wild type) [49]. The consideration of using targeted biological agents in tumours that lack RAS/BRAF mutations in left-sided CRC. Research has shown that the site of the primary tumour influences the effectiveness of anti-epidermal growth factor receptor (EGFR) agents; we avoid the use of an anti-EGFR agent in right-sided primary tumours, even if RAS/BRAF wild type [25,50]. Evidence from the EPOC trial highlights that in 272 patients with resectable hepatic metastases of KRAS wild-type mCRC were given FOLFOX with or without cetuximab, pre-operatively and post-operatively for 12 weeks, and it is clear that the addition of cetuximab was associated with significantly worse progression-free survival (PFS) (15.5 versus 22.2 months) [22,51,52].

### 3.6. Patients with Initially Unresectable Metastases

There is a necessity to utilize chemotherapy and targeted biological therapies in patients with unresectable disease in order to downstage their condition. With combination conversional therapy, there is a 10–15% likelihood of converting unresectable CRLM into resectable CRLM. Patients with unresectable CRLM often undergo prolonged periods of chemotherapeutic and biological therapies. Prolonged exposure to such therapies can trigger perioperative and post-operative complications, including liver toxicity and hepatic dysfunction [53,54,55]. The EPOC trial reported ~12–33% of patients with initial unresectable CRLM who were converted to resectable to allow surgical intervention to achieve a complete resection (R0) [22].

With the combined chemotherapeutics and targeted biotherapies, the 5-year survival rates average 30% to 35%, which is significantly better when patients just used chemotherapy alone; the 5-year survival rates on average were 10% to 11% [56]. The benefit from the addition of a biologic agent is controversial but, in the CRYSTAL [57,58], DEEPER [59], and OPUS [51,60] trials both have shown modestly improved resectability rates when using chemotherapeutics plus targeted biological agents vs. chemotherapy alone (3.7% (chemo. alone) vs. 7% (chemo. plus, targeted biological agents) and from 2.4% (chemo. alone) vs. 4.7% (chemo. plus targeted biological agents) respectively [57,58]. This, as a result, increases the number of patients potentially eligible for resection and improve outcomes. With mCRC/CRLM with KRAS mutations and KRAS wild-type in the OPUS trial, the use of FOLFOX plus cetuximab increased resectability compared with just chemotherapy alone (4% vs. 10%) [61,62]. EPOC trial data recommended complete avoidance of a combination of an anti-EGFR agent plus oxaliplatin, even in patients with left-sided tumours. The benefit of anti-EGFR agent plus oxaliplatin is marginal at best, and the complications associated with prolonged therapeutics exposure outweighs its marginal and unestablished benefits [63].

### 3.7. Adjuvant Treatment When Resectable

Similar to neoadjuvant therapy in resectable patients, there are no clear guidelines or best post-operative strategy. However, common practice is that after confirmed complete resection, most centres deliver a total of a six-month course of systemic chemotherapy, including chemotherapeutics delivered as neoadjuvant therapy. The frontrunners of agents used are FOLFOX alone, or CAPOX can be used. With regards to the use of chemotherapeutics and targeted biological agents in the adjuvant setting after resection of CRLM, the addition of bevacizumab with conventional chemotherapy did not prolong survival but induced biliary toxicity [64,65,66].

## 4. Biotherapies and Their Action Modes

Biological therapies are credited with increasing median overall survival in colorectal cancer, and it is a vastly active area of cancer research with the sole objective of improving patient outcomes in conjunction with co-existing therapies. Biotherapies are a form of targeted therapeutics with a biological focus on a colorectal cancer cell, key for cancer cell function including cell surface markers and receptors, assisting in the host’s immune response by (1) inducing the immune system to target cancer cells and (2) targets the cancer cells, controlling the on or off cell signals that assist the CRC cells in eluding the immune system cells (e.g., immune checkpoint inhibitors which target specific chemical receptors on cancer cells, blocking the signals the cancer cells send to suppress the immune system [67,68,69]. There are a number of types of biological therapies that extend to: adoptive cell transfer, immunomodulators, chimeric antigen (CAR) T cell therapy, monoclonal antibodies, cancer vaccines and immune checkpoint inhibitors.

### 4.1. Immunotherapy and Adoptive Cell Transfer

Immunotherapy has become the frontrunner treatment option for the management of several malignancies that have become unresponsive and insensitive to chemo-radiotherapy and thereby prevents principal pathways of treatment failure, which ultimately leads to metastasis. The mainstay treatment option for CRC showed minimal improvement in the 5-year survival rate and prevented re-occurrence after apparent complete resection even with relapses that followed a complete surgical hepatic resection. The function of immunotherapies acts on blocking tumourigenic interactions within the cancer-stromal microenvironment and inhibiting the action of secreted cytokines and cell surface receptors these cytokines bind to, which are involved in tumorigenesis [70,71].

Immunotherapies facilitated the blocking of cytokine signalling to target receptor, CCR5, which prevents the progression of the tumour microenvironment via the inhibition of the interactions myeloid cells (MDSCs) and T cells and therefore achieved clinical benefits among patients with advanced/mCRC [72]. The liver’s microenvironment is immunosuppressive in nature and aids the development of metastatic regions within the hepatic microenvironment. The presence of MDSCs has proven a key mediator for metastases. The effectiveness of CAR-T cells engineered against hepatic metastases can be weakened by the presence of MDSCs [73].

Hepatic MDSCs express PD-L1 (Programmed death-ligand 1) which binds onto the PD-1 on the T cell surface promotes cell death, and prevents the activation and differentiation of T cells and, therefore, unable to action its anti-tumour function [74,75]. By reducing MDSC accumulation within the liver via PD-L1 blockade from immunotherapy like CAR-T cell therapy, you are then able to promote and achieve antitumor responses. Moreover, the blockade of molecules involved in MDSC biology and function augmented the efficiency of adoptive cell therapy against CRC metastases. Adoptive cell therapy (ACT) using tumour-infiltrating lymphocyte (TILs) has shown significant efficacy in patients with metastatic melanoma and might be a feasible option for most CRC patients with liver metastases [76,77].

### 4.2. Targeted Biological Therapy

Targeted therapies can work on cancerous cells by directly inhibiting cell proliferation, differentiation, and migration [78]. Targeted biological therapies have been a long-standing concept initially thought to expand on current cancer treatment regimes. The targets are the functional components of tissues and tumours, including blood vessels and immune cells [79]. Targeted biological therapy for mCRC has become an important aspect of the “conversional” management of unresectable CRLM. Over time, research has allowed us to understand tumour biology and to understand the mechanism of their targets. The two main mechanisms included the angiogenesis pathway (inhibited by bevacizumab) and epidermal growth factor receptors (EGFRs: cetuximab and panitumumab) [80]. EPOC trial provides evidence that adding targeted biological therapeutics with chemotherapeutics causes an increase in the number of candidates eligible for surgery. In general, response rates appear to be highest with the EGFRIs; therefore, these agents may potentially also lead to greater resection rates.

The tumour microenvironment, including local blood vessels and immune cells, might also be altered by targeted drugs to impede tumour growth and enact stronger immune surveillance and attack. For targets outside cells, such as cell surface receptors or membrane-bound sites, monoclonal antibodies or therapeutic antibodies can recognize and bind them to directly regulate downstream cell cycle progression and cell death [81]. In addition, certain monoclonal antibodies work on cells other than cancer cells, such as immune cells, which helps to manipulate the immune system to attack human cancer.

As mentioned earlier, intrinsic or acquired resistance may exist and identifying these potential resistances to targeted therapies are of high importance: (1) Predicting resistance to the Epidermal Growth Factor Receptor (EGFR)-targeted monoclonal antibodies: different studies have demonstrated that combined detection of *NRAS*, *PTEN and PI3K* (especially exon 20) genes status in wild-type *KRAS* CRC patients, can identify patients who are more prone to not respond to anti-EGFR therapies [82] and (2) identifying intrinsic and acquired resistance to drugs targeting the EGFR-RAS-RAF pathway in CRC, in addition to identifying potential biomarkers for resistance of tumours that are dependent on MEK signalling: the PI3K/mTOR pathway may be involved in the resistance to MEK inhibition in CRC but also in the RAS-RAF-MEK-MAPK pathway [83].

### 4.3. Future of Biotherapies for Liver Metastasis in Colorectal Cancer

#### 4.3.1. Immunotherapy

Colorectal liver metastases remain associated with poor overall prognosis, even with surgical resection of the tumour at its primary site [84]. As with other cancers, immunosuppressive micro-environments within the liver support tumourigenesis to develop hepatic metastases [85]. For instance, myeloid-derived stem cells (MDSC’s) residing within the liver micro-environment can express PD-L1, which inhibit the activation and proliferation of T cells by interacting with PD-1 on T cells. Owing to the complex interaction between the immune system and tumour cells, immunotherapy has achieved a certain [86] degree of success in the treatment of advanced solid tumours in recent years [87]. Principally, immunotherapy aims to enhance or amplify the anti-tumour response by the patient’s own immune system by augmenting the innate immunity and anti-tumour function of T cells and by targeting immunosuppressive tumour-associated macrophages [88]. Broadly, immunotherapies include: (i) immune checkpoint inhibitors (ICIs); (ii) cancer vaccines; and (iii) other biotherapeutics such as chimeric antigen receptor (CAR) T cells. These therapies are effective in treating a variety of cancers [86], including mCRC, especially mCRC with deficient DNA mismatch repair (dMMR) genes [89].

#### 4.3.2. Immunotherapy in Colorectal Liver Metastasis

Several immunotherapy agents have been being evaluated in prospective randomized clinical trials (RCT) in CLM [90]. Firstly, RCT’s on pembrolizumab demonstrated improvement in progression-free survival rates compared to no treatment in MSI-H CRC’s (11% vs. 0%) [91]. Compared to MSI cancers, MSS cancers are less sensitive to immunotherapy alone. This is because dMMR CRCs are prone to be heavily infiltrated by CD8+ T cells and highly expressed immune checkpoint molecules, including PD-L1 [92]. In dMMR CRC and pMMR CRC, the disease control rate at 12 weeks was 90% and 11%, respectively [93]. This might partly explain why anti-PD-1/PD-L1 therapies are correlated to antitumor responses in MSI-H or dMMR mCRC. Therefore, novel combination approaches are being investigated to enhance therapeutic efficiency in patients who might be sensitive to combination treatments [94]. Secondly, the use of nivolumab in conjunction with or without ipilimumab in dMMR or MSI-H mCRC have been assessed in the phase II CheckMate 142 RCT. Preliminary data revealed an objective response rate (ORR) and disease control rate of 31% and 69%, respectively. When ipilimumab was added to nivolumab, the ORR and disease control rates of 41% and 78%, respectively, were noted [95]. Nivolumab has been approved for patients harbouring dMMR/MSI-H mCRC who experience disease progression following treatment of fluoropyrimidine, oxaliplatin, and irinotecan [96]. However, response rates varied in CRC with distinct genomic alterations [97].

Thirdly, adoptive cell therapy (ACT) has shown improvement in survival in patients with metastatic melanoma. Similar responses have been observed in individual patients with CRC after ACT [98,99]. CAR therapy aims to improve NK cell tumour response. A recent study confirmed that CAR-NK cells could recognize tumour cells and exhibit anti-tumour effects in metastatic CLM patients [100]. This is an achievable way to combat liver metastasis by using new techniques developed in recent years. Because it can be harvested aseptically and does not contaminate the intestinal flora, liver metastasis may be an ideal source of tumour-infiltrative lymphocytes for the treatment of ACT in CRC patients [97].

## 5. Beyond the Treatment of CRC and Its Progression: Prevention of CRC Occurrence and Metastatic Recurrence Is of High Importance

The key to primary prevention of CRC occurrence is how successful our methods of educating the population on highlighting high-risk factors, dietary advice and promoting healthy lifestyles. These include identifying patients that are genetically at risk or are in high-risk families (Families with Familial Adenomatous Polyposis [FAP], Hereditary Non-Polyposis Colorectal Cancer [HNPCC or Lynch syndrome]) and identifying family history for the presence of previous CRC or polyps [101]. Genetic mutations play an integral role in CRC. Recent studies have highlighted important germline mutations at different locations of a number of target genes [102]. Patients with CRC will often harbour potentially pathogenic germline mutations. Patients can have germline mutations in non-mismatch repair genes, including *MUTHY*, *RAD50*, *TP53*, and a rarer cohort of other genes [103]. Both young adults or those who have been diagnosed with early-onset CRC and mCRC often have comparable mutations in *TP53*, *APC*, *KRAS*, *SMAD4*, *BRAF*, *FAF1 and BRCA2* genes but varied tumour mutation burden (TMB), with a higher TMB linked to early-onset CRC [104]. On a cellular pathway level, genetic mutations in cellular pathways WNT and TGF-Β pathways are also associated with unfavorable OS in CRC. Therefore, screening and early detection is important in improving OS [105].

Early detection and screening have played an important role also in the CRC occurrence prevention of disease’s progression; by means of patient acknowledgement and awareness of symptoms, it aids in the reduction of time from diagnosis to commencement of treatment [106]. National early detection initiatives have also reduced the stigma associated with the perception of CRC and thereby have increased population engagement and CRC detection [107]. In regards to prevention of CRC metastatic recurrence, routine surveillance following national guidelines, but also neoadjuvant and adjuvant therapeutics to downstage disease and reduce metastatic disease, all these elements assist in the survivorship in affected patients [108].

### 5.1. Environment, Diet, Lifestyle, Microbiome, and Immune System Together Influence Pathogenic Mechanisms of Colorectal Carcinogenesis

#### 5.1.1. Dietary Advice

Diet has a proven link with the risk of developing CRC. Dietary elements such as foods with high dietary fats, red meats, high consumption of alcohol are all associated with increasing the risk of promoting CRC [109]. In contrast, the consumption of fruits, vegetables, high fibre foods, foods rich in vitamin B6 and D are associated with lowering the risk of CRC development. A recent systematic review by Siegel et al. [110] supports that the integration of healthy eating and physical activity substantially lowers the risks of developing CRC.

#### 5.1.2. Pre-Existing Conditions

Conditions including inflammatory bowel disease (IBD), primarily Ulcerative colitis (UC) more than Crohn’s disease (CD), hypertension, chronic kidney disease, hyperlipidaemia and obesity and others are linked to increased risk of the development of CRC. There is a clear association between ulcerative colitis, the chronicity of the disease and colonic neoplasia [111]. The presence of pancolitis in UC increases the risk of CRC by up to 15 times when compared to the average national risk. UC patients also undergo rigorous screening for polyps. CD sufferers who have extensive and prolonged occurrences of pancolitis have a relative risk to chronic pancolitis similar to that of UC patients [112]. Young adult and paediatric patients who have been diagnosed with malignancy and treated with abdominopelvic radiation has a direct correlation to and increases the relative risk of developing CRC. Co-morbidities, including diabetes mellitus (DM), obesity, hypertension and chronic kidney disease, is associated with an elevated relative risk of CRC [113].

#### 5.1.3. Gut Microbiome Immunology and CRC

The intestinal microbiota is composed of a large, varied array of micro-organisms. It is estimated that the human intestinal microbiota is composed of 10^13^ to 10^14^ microbes [114]. These microbes play an essential role in establishing ‘gut homebiosis’ and preventing gut dysbiosis, which drives disease and pathology. Recent articles have identified that micro-organisms such as *Streptococcus Bovis*, *enterotoxigenic Bacteroides fragilis*, *Fusobacterium nucleatum*, *Enterococcus faecalis*, *Escherichia coli*, *and Peptostreptococcus anaerobius* as CRC candidate pathogens [115]. The composition and balance in the type of micro-organisms is the key to reducing the risk of developing a range of conditions and diseases. Alterations in the composition of the gut microbiota shape and alter the intestinal epithelium and its metabolic activity. *Streptococcus Bovis*, which is normally found in the gastrointestinal tract, can act as an early indicator for CRC [116] if increasing concentrations of this microbe are detected. Microbes such as enterotoxigenic *Bacteroides fragilis*, *Fusobacterium nucleatum*, *Enterococcus faecalis*, *Escherichia coli*, *and Peptostreptococcus anaerobius* are found in increased quantities when compared to healthy individuals’ intestinal microbiota [117]. Even in the presence of colorectal adenomas, we see a considerable change in the concentration of the aforementioned microbes in the gut microbiota. There is a capability of being able to quantify and detect the microbiota alterations as alternative biomarkers for CRC detection [118].

### 5.2. Molecular Pathology Research towards the Environment, Lifestyle, Microbiome, Immunity for Prevention, Treatment and Clinical Outcomes Regarding CRC Management

The mechanisms in which these microbiota drive the pathogenesis of CRC are linked to an inflammation theory. This corresponds to the secretion of toxins by certain microbes, which in turn damages intestinal epithelium and epithelial cell DNA, thus initiating the CRC process [119]. With the presence of dysbiosis due to the increase in specific gut microbiota, there is an increase in causative microbes, which triggers chronic inflammation and consequently tumorigenesis [120]. There is a loss of the intestinal mucosal barrier, an important factor in dysbiosis and inflammation propagation. The loss of the mucosal layer results in the intestinal layer becoming highly permeable to the gut microbiota, which initiates worsening inflammation and colitis [121]. Toll-Like Receptors (TLRs) and Pattern recognition receptors (PRRs) are activated by components of the microbes of the gut flora, including LPS (liposaccharide) found in the cell walls of microbiota [122]. Downstream signalling and activation occur and drive the production of inflammatory cytokines such as IL-17, IL22 and Il-23; this then eventually promotes tumour cell proliferation by activation nuclear factor-κB (NFκB) and STAT3 signalling pathway and eventually CRC development [123].

## 6. Conclusions

In summary, treatment of mCRC, especially CLM, is challenging because of its immunosuppressive microenvironment. Therefore, immunotherapy may be a novel therapeutic strategy for this cohort of patients. Despite increasing clinical data regarding the therapeutic role of immunotherapy among dMMR or MSI-H mCRC, evidence for most patients harbouring pMMR or MSS tumours still do not benefit from immunotherapeutic agents [124]. This may be explained by the collective inhibitory impact of multiple networks on effector immune cells to enable CRC to develop and form metastases within the tumour micro-environment [125]. This necessitates further research to develop novel therapeutic approaches or identify biomarkers for personalized modulation of the tumour micro-environment to reverse immunosuppression, thus improving clinical outcomes [126]. Specifically, the immune cells in the tumour micro-environment, together with the soluble factors, might also be potential targets to treat CLM. Outside of the discovery of new immunotherapy targets, further directions should be focused on relieving CLM from the inhibitory networks and activation hurdles constituting the TME by combining immunotherapies with other therapies.

## Figures and Tables

**Table 1:** Combined Chemotherapy Regimens.

Names of Combined Chemotherapy Regimens	Components of Combined Chemotherapy
FOLFOX	Folinic Acid, Fluorouracil and Oxaliplatin.
FOLFORI	5-Fluorouracil and High-Dose Leucovorin
FOLFIRI	Folinic Acid, Fluorouracil and Irinotecan
FOLFOXIRI	Folinic Acid, Fluorouracil and Oxaliplatin.
CAPOX or XELOX	Oxaliplatin and Capecitabine

**Table 2:** Types of anticancer therapies.

Types of Therapy	Definition	Examples of Type of Therapy
“Classical” Cytotoxic Chemotherapies	Therapies that can be delivered intravenously or orally. It can be given during the neoadjuvant, adjuvant or palliative setting and can be given either systemically or regionally.	FOLFOX (also known as Oxaliplatin de Gramont or OxMdG, which means modified Oxaliplatin de Gramont) (Folinic acid, fluorouracil and oxaliplatin) FOLFORI (5-Fluorouracil and High-Dose Leucovorin) FOLFIRI (Folinic acid, fluorouracil and irinotecan) FOLFOXIRI (folinic acid, 5-fluorouracil, oxaliplatin and irinotecan) CAPOX (capecitabine plus oxaliplatin) or XELOX (xeloda^®^ = capecitabine plus oxaliplatine)
Targeted Therapies	Therapies that target specific molecules, including receptors, proteins, genes which impair the development and propagation of tumour growth.	Epidermal Growth Factor Receptor Inhibitor: Cetuximab and Panitumumab Tyrosine Kinase Inhibitor: Regorafenib *BRAF* Inhibitors: Encorafenib
Anti-angiogenic Therapies	Therapies against which targets the protein (VEGF) that promotes vessel development and growth in order to facilitate transportation of nutrients to the tumour in order for tumour growth.	Bevacizumab Ramucirumab Ziv-aflibercept
Biotherapies	Therapies that utilize and facilitate a patient’s own immune system in recognizing and killing present cancer cells, e.g., immunotherapies.	Monoclonal Antibodies CAR T-Cell Therapy Immune Checkpoint Inhibitors Cancer Vaccines Immunomodulators

## Data Availability

Data and materials are at the disposal of the journal.

## References

[1] Saridaki Z., Souglakos J., Papamichael D., Audisio R. (2013). Genetic Alterations in Colorectal Cancer in Older Patients. Management of Colorectal Cancers in Older People.

[2] Rivlin N., Brosh R., Oren M., Rotter V. (2011). Mutations in the p53 Tumor Suppressor Gene: Important Milestones at the Various Steps of Tumorigenesis. Genes Cancer.

[3] Dhirendra K.S., Dwight V.N., McCormick F. (2017). RAS Proteins and Their Regulators in Human Disease. Cell.

[4] Guo Y., Pan W., Liu S., Shen Z., Xu Y., Hu L. (2020). ERK/MAPK signalling pathway and tumorigenesis (Review). Exp. Ther. Med..

[5] Cheng Y., Tian H. (2017). Current Development Status of MEK Inhibitors. Molecules.

[6] Maverakis E., Tran K., Cheng M., Mitra A., Ogawa H., Shi V., Olney L., Kloxin A. (2015). MEK inhibitors and their potential in the treatment of advanced melanoma: The advantages of combination therapy. Drug Des. Dev. Ther..

[7] Samir B., Arnab G. (2020). Colorectal Liver Metastasis: Current Concepts. Indian J. Surg..

[8] Martin J., Petrillo A., Smyth E.C., Shaida N., Khwaja S., Cheow H.K., Duckworth A., Heister P., Praseedom R., Jah A. (2020). Colorectal liver metastases: Current management and future perspectives. World J. Clin. Oncol..

[9] Voizard N., Cerny M., Assad A., Billiard J.-S., Olivié D., Perreault P., Kielar A., Do R.K.G., Yokoo T., Sirlin C.B. (2019). Assessment of hepatocellular carcinoma treatment response with LI-RADS: A pictorial review. Insights Imaging.

[10] Tamandl D., Mang T., Ba-Ssalamah A. (2020). Imaging of colorectal cancer—The clue to individualized treatment. Innov. Surg. Sci..

[11] Venook A.P., Curley S.A. (2020). Management of potentially resectable colorectal cancer liver metastases. World J. Gastrointest. Surg..

[12] Chow F.C.-L., Chok K.S.-H. (2019). Colorectal liver metastases: An update on multidisciplinary approach. World J. Hepatol..

[13] Adam R., de Gramont A., Figueras J., Kokudo N., Kunstlinger F., Loyer E., Poston G., Rougier P., Rubbia-Brandt L., Sobrero A. (2015). Managing synchronous liver metastases from colorectal cancer: A multidisciplinary international consensus. Cancer Treat. Rev..

[14] Dhir M., Sasson A.R. (2016). Surgical Management of Liver Metastases from Colorectal Cancer. J. Oncol. Pr..

[15] Ono K., Abe T., Oshita A., Sumi Y., Yano T., Okuda H., Kurayoshi M., Kobayashi T., Ohdan H., Noriyuki T. (2021). Efficacy of upfront hepatectomy without neoadjuvant chemotherapy for resectable colorectal liver metastasis. World J. Surg. Oncol..

[16] Paulatto L., Burgio M.D., Sartoris R., Beaufrère A., Cauchy F., Paradis V., Vilgrain V., Ronot M. (2020). Colorectal liver metastases: Radiopathological correlation. Insights Imaging.

[17] Stevenson H.L., Prats M.M., Sasatomi E. (2017). Chemotherapy-induced Sinusoidal Injury (CSI) score: A novel histologic assess-ment of chemotherapy-related hepatic sinusoidal injury in patients with colorectal liver metastasis. BMC Cancer.

[18] Pfeiffer P., Gruenberger T., Glynne-Jones R. (2018). Synchronous liver metastases in patients with rectal cancer: Can we establish which treatment first?. Ther. Adv. Med. Oncol..

[19] Al Bandar M.H., Kim N.K. (2017). Current status and future perspectives on treatment of liver metastasis in colorectal cancer (Re-view). Oncol. Rep..

[20] Benoist S., Brouquet A., Penna C., Julié C., Hajjam M.E., Chagnon S., Mitry E., Rougier P., Nordlinger B. (2006). Complete response of colorectal liver metastases after chemotherapy: Does it mean cure?. J. Clin. Oncol..

[21] Symonds L.K., Cohen S.A. (2019). Use of perioperative chemotherapy in colorectal cancer metastatic to the liver. Gastroenterol. Rep..

[22] Primrose J., Falk S., Finch-Jones M., Valle J., O’Reilly D., Siriwardena A., Hornbuckle J., Peterson M., Rees M., Iveson T. (2020). Systemic chemotherapy with or without cetuximab in patients with resectable colorectal liver metas-tasis (New EPOC): Long-term results of a multicentre, randomized, controlled, phase 3 trial. Lancet Oncol..

[23] Wensink E., Bond M., Kucukkose E., May A., Vink G., Koopman M., Kranenburg O., Roodhart J. (2021). A review of the sensitivity of metastatic colorectal cancer patients with deficient mismatch repair to stand-ard-of-care chemotherapy and monoclonal antibodies, with recommendations for future research. Cancer Treat. Rev..

[24] Poston G., Adam R., Byrne B., Esser R., Malik H., Wasan H., Xu J. (2017). The role of cetuximab in converting initially unresectable colorectal cancer liver metastases for resection. Eur. J. Surg. Oncol..

[25] Xie Y.-H., Chen Y.-X., Fang J.-Y. (2020). Comprehensive review of targeted therapy for colorectal cancer. Signal Transduct. Target. Ther..

[26] Ichida H., Mise Y., Ito H., Ishizawa T., Inoue Y., Takahashi Y., Shinozaki E., Yamaguchi K., Saiura A. (2019). Optimal indication criteria for neoadjuvant chemotherapy in patients with resectable colorectal liver metas-tases. World J. Surg. Oncol..

[27] De Greef K., Rolfo C., Russo A., Chapelle T., Bronte G., Passiglia F., Coelho A., Papadimitriou K., Peeters M. (2016). Multisciplinary management of patients with liver metastasis from colorectal cancer. World J. Gastroenterol..

[28] Sabanathan D., Eslick G.D., Shannon J. (2016). Use of Neoadjuvant Chemotherapy Plus Molecular Targeted Therapy in Colorectal Liver Metastases: A Systematic Review and Meta-analysis. Clin. Color. Cancer.

[29] Villaruz L.C., Socinski M.A. (2013). The clinical viewpoint: Definitions, limitations of RECIST, practical considerations of measure-ment. Clin. Cancer Res..

[30] Aykan N.F., Özatlı T. (2020). Objective response rate assessment in oncology: Current situation and future expectations. World J. Clin. Oncol..

[31] Mahvi D.A., Liu R., Grinstaff M.W., Colson Y.L., Raut C.P. (2018). Local Cancer Recurrence: The Realities, Challenges, and Opportunities for New Therapies. CA A Cancer J. Clin..

[32] Tohme S., Simmons R.L., Tsung A. (2017). Surgery for Cancer: A Trigger for Metastases. Cancer Res..

[33] Engstrand J., Nilsson H., Strömberg C., Jonas E., Freedman J. (2018). Colorectal cancer liver metastases—A population-based study on incidence, management and survival. BMC Cancer.

[34] Vatandoust S., Price T.J., Karapetis C. (2015). Colorectal cancer: Metastases to a single organ. World J. Gastroenterol..

[35] Lam V.W.T., Laurence J.M., Pang T., Johnston E., Hollands M.J., Pleass H.C.C., Richardson A.J. (2014). A systematic review of a liver-first approach in patients with colorectal cancer and synchronous colorec-tal liver metastases. HPB.

[36] Sahlmann C.-O., Homayounfar K., Niessner M., Dyczkowski J., Conradi L.-C., Braulke F., Meller B., Beißbarth T., Ghadimi B.M., Meller J. (2017). Repeated adjuvant anti-CEA radioimmunotherapy after resection of colorectal liver metastases: Safety, feasibility, and long-term efficacy results of a prospective phase 2 study. Cancer.

[37] Creasy J.M., Sadot E., Koerkamp B.G., Chou J.F., Gonen M., Kemeny N.E., Balachandran V.P., Kingham T.P., DeMatteo R.P., Allen P.J. (2018). Actual 10-year survival following hepatic resection of colorectal liver metastases: What factors preclude cure?. Surgery.

[38] Wang L.-J., Wang H.-W., Jin K.-M., Li J., Xing B.-C. (2020). Comparison of sequential, delayed and simultaneous resection strategies for synchronous colorectal liver metastases. BMC Surg..

[39] Du Pasquier C., Roulin D., Bize P., Sempoux C., Rebecchini C., Montemurro M., Schäfer M., Halkic N., Demartines N. (2020). Tumor response and outcome after reverse treatment for patients with synchronous colorectal liver metastasis: A cohort study. BMC Surg..

[40] Feo L., Polcino M., Nash G.M. (2017). Resection of the Primary Tumor in Stage IV Colorectal Cancer: When Is It Necessary?. Surg. Clin. N. Am..

[41] Kuipers E.J., Grady W.M., Lieberman D., Seufferlein T., Sung J.J., Boelens P.G., van de Velde C.J.H.T.W. (2015). Colorectal Cancer. Nat. Rev. Dis. Prim..

[42] Basso M., Dadduzio V., Ardito F., Lombardi P., Strippoli A., Vellone M., Orlandi A., Rossi S., Cerchiaro E., Cassano A. (2016). Conversion Chemotherapy for Technically Unresectable Colorectal Liver Metastases. Medicine.

[43] Poultsides G.A., Bao F., Servais E.L., Hernandez-Boussard T., DeMatteo R.P., Allen P.J., Fong Y., Kemeny N.E., Saltz L.B., Klimstra D.S. (2012). Pathologic response to pre-operative chemotherapy in colorectal liver metastases: Fibrosis, not necrosis, predicts outcome. Ann. Surg. Oncol..

[44] Langella S., Ardito F., Russolillo N., Panettieri E., Perotti S., Mele C., Giuliante F., Ferrero A. (2019). Intraoperative Ultrasound Staging for Colorectal Liver Metastases in the Era of Liver-Specific Magnetic Resonance Imaging: Is It Still Worthwhile?. J. Oncol..

[45] Joo I. (2015). The role of intraoperative ultrasonography in the diagnosis and management of focal hepatic lesions. Ultrasonography.

[46] McKeown E., Nelson D., Johnson E.K., Maykel J.A., Stojadinovic A., Nissan A., Avital I., Brücher B., Steele S.R. (2014). Current Approaches and Challenges for Monitoring Treatment Response in Colon and Rectal Cancer. J. Cancer.

[47] Van Cutsem E., Cervantes A., Adam R., Sobrero A., van Krieken J., Aderka D., Aguilar E.A., Bardelli A., Benson A., Bodoky G. (2016). ESMO consensus guidelines for the management of patients with metastatic colorectal cancer. Ann. Oncol..

[48] De’Angelis N., Baldini C., Brustia R., Pessaux P., Sommacale D., Laurent A., Le Roy B., Tacher V., Kobeiter H., Luciani A. (2020). Surgical and regional treatments for colorectal cancer metastases in older patients: A systematic review and meta-analysis. PLoS ONE.

[49] Ikoma N., Raghav K., Chang G. (2017). An Update on Randomized Clinical Trials in Metastatic Colorectal Carcinoma. Surg. Oncol. Clin. N. Am..

[50] Yokota T. (2012). Are KRAS/BRAF Mutations Potent Prognostic and/or Predictive Biomarkers in Colorectal Cancers?. Anticancer Agents Med. Chem..

[51] Yang Y.F., Wang G.Y., He J.L., Wu F.P., Zhang Y.N. (2017). Overall survival of patients with KRAS wild-type tumor treated with FOLFOX/FORFIRI±cetuximab as the first-line treatment for metastatic colorectal cancer A meta-analysis. Medicine.

[52] Chan G., Chee C.E. (2020). Perioperative Chemotherapy for Liver Metastasis of Colorectal Cancer. Cancers.

[53] Ma R., Li T. (2021). Conversion therapy combined with individualized surgical treatment strategy improves survival in patients with colorectal cancer liver metastases. Int. J. Clin. Exp. Pathol..

[54] Villard C., Habib M., Nordenvall C., Nilsson P., Jorns C., Sparrelid E. (2021). Conversion therapy in patients with colorectal liver metastases. Eur. J. Surg. Oncol..

[55] Ismaili N. (2011). Treatment of colorectal liver metastases. World J. Surg. Oncol..

[56] Vogel A., Kirstein M.M. (2018). First-line molecular therapies in the treatment of metastatic colorectal cancer—A literature-based review of phases II and III trials. Innov. Surg. Sci..

[57] Van Cutsem E., Köhne C.-H., Láng I., Folprecht G., Nowacki M.P., Cascinu S., Shchepotin I., Maurel J., Cunningham D., Tejpar S. (2011). Cetuximab Plus Irinotecan, Fluorouracil, and Leucovorin As First-Line Treatment for Metastatic Colorectal Cancer: Updated Analysis of Overall Survival According to Tumor KRAS and BRAF Mutation Status. J. Clin. Oncol..

[58] Van Cutsem E., Köhne C.-H., Hitre E., Zaluski J., Chien C.-R.C., Makhson A., D’Haens G., Pintér T., Lim R., Bodoky G. (2009). Cetuximab and Chemotherapy as Initial Treatment for Metastatic Colorectal Cancer. N. Engl. J. Med..

[59] Tsuji A., Ohori H., Yamaguchi T., Matsuura M., Nishioka A., Makiyama A., Noura S., Kochi M., Sagawa T., Kotaka M. (2021). Safety analysis of the randomized phase II study of FOLFOXIRI plus cetuximab versus FOLFOXIRI plus bevacizumab as the first-line treatment in metastatic colorectal cancer with RAS wild-type tumors: The DEEPER trial (JAC-CRO CC-13). J. Clin. Oncol..

[60] Bokemeyer C., Bondarenko I., Hartmann J.T., de Braud F., Schuch G., Zubel A., Celik I., Schlichting M., Koralewski P. (2011). Efficacy according to biomarker status of cetuximab plus FOLFOX-4 as first-line treatment for metastatic colorectal cancer: The OPUS study. Ann. Oncol..

[61] Sullivan K.M., Kozuch P.S. (2011). Impact of KRAS Mutations on Management of Colorectal Carcinoma. Pathol. Res. Int..

[62] Sotelo M., García-Paredes B., Aguado C., Sastre J., Díaz-Rubio E. (2014). Role of cetuximab in first-line treatment of metastatic colorectal cancer. World J. Gastroenterol..

[63] Chen D., Li L., Zhang X., Gao G., Shen L., Hu J., Yang M., Liu B., Qian X. (2018). FOLFOX plus anti-epidermal growth factor receptor (EGFR) monoclonal antibody (mAb) is an effective first-line treatment for patients with RAS-wild left-sided metastatic colorectal cancer: A meta-analysis. Medicine.

[64] Comunanza V., Bussolino F. (2017). Therapy for Cancer: Strategy of Combining Anti-Angiogenic and Target Therapies. Front. Cell Dev. Biol..

[65] Ohhara Y., Fukuda N., Takeuchi S., Honma R., Shimizu Y., Kinoshita I., Dosaka-Akita H. (2016). Role of targeted therapy in metastatic colorectal cancer. World J. Gastrointest. Oncol..

[66] Shuford R.A., Cairns A.L., Moaven O. (2020). Precision Approaches in the Management of Colorectal Cancer: Current Evidence and Latest Advancements Towards Individualizing the Treatment. Cancers.

[67] Markman J.L., Shiao S.L. (2015). Impact of the immune system and immunotherapy in colorectal cancer. J. Gastrointest. Oncol..

[68] Lynch D., Murphy A. (2016). The emerging role of immunotherapy in colorectal cancer. Ann. Transl. Med..

[69] Zhang B., Cheng P. (2020). Improving antitumor efficacy via combinatorial regimens of oncolytic virotherapy. Mol. Cancer.

[70] Matsuoka H., Morise Z., Tanaka C., Hayashi T., Ikeda Y., Maeda K., Masumori K., Koide Y., Katsuno H., Tanahashi Y. (2019). Repeat hepatectomy with systemic chemotherapy might improve survival of recurrent liver metastasis from colorectal cancer—A retrospective observational study. World J. Surg. Oncol..

[71] Lintoiu-Ursut B., Tulin A., Constantinoiu S. (2015). Recurrence after hepatic resection in colorectal cancer liver metastasis—Review article. J. Med. Life.

[72] Gabrilovich D.I., Nagaraj S. (2009). Myeloid-derived suppressor cells as regulators of the immune system. Nat. Rev. Immunol..

[73] De Cicco P., Ercolano G., Ianaro A. (2020). The New Era of Cancer Immunotherapy: Targeting Myeloid-Derived Suppressor Cells to Overcome Immune Evasion. Front. Immunol..

[74] Bocanegra A., Blanco E., Fernandez-Hinojal G., Arasanz H., Chocarro L., Zuazo M., Morente P., Vera R., Escors D., Kochan G. (2020). PD-L1 in Systemic Immunity: Unraveling Its Contribution to PD-1/PD-L1 Blockade Immunotherapy. Int. J. Mol. Sci..

[75] Yang Y., Li C., Liu T., Dai X., Bazhin A.V. (2020). Myeloid-Derived Suppressor Cells in Tumors: From Mechanisms to Antigen Specificity and Microenvironmental Regulation. Front. Immunol..

[76] Saint-Jean M., Knol A.-C., Volteau C., Quéreux G., Peuvrel L., Brocard A., Pandolfino M.-C., Saiagh S., Nguyen J.-M., Bedane C. (2018). Adoptive Cell Therapy with Tumor-Infiltrating Lymphocytes in Advanced Melanoma Patients. J. Immunol. Res..

[77] Jiménez-Reinoso A., Nehme-Álvarez D., Domínguez-Alonso C., Álvarez-Vallina L. (2021). Synthetic TILs: Engineered Tu-mor-Infiltrating Lymphocytes with Improved Therapeutic Potential. Front. Oncol..

[78] Yang L., Shi P., Zhao G., Xu J., Peng W., Zhang J., Zhang G., Wang X., Dong Z., Chen F. (2020). Targeting cancer stem cell pathways for cancer therapy. Signal Transduct. Target. Ther..

[79] Lugano R., Ramachandran M., Dimberg A. (2020). Tumor angiogenesis: Causes, consequences, challenges and opportunities. Cell. Mol. Life Sci..

[80] Zhao B., Wang L., Qiu H., Zhang M., Sun L., Peng P., Yu Q., Yuan X. (2016). Mechanisms of resistance to anti-EGFR therapy in colorectal cancer. Oncotarget.

[81] Hallam S., Escorcio-Correia M., Soper R., Schultheiss A., Hagemann T. (2009). Activated macrophages in the tumour microenvi-ronment—Dancing to the tune of TLR and NF-κB. J. Pathol..

[82] De Roock W., Claes B., Bernasconi D., De Schutter J., Biesmans B., Fountzilas G., Kalogeras K.T., Kotoula V., Papamichael D., Laurent-Puig P. (2010). Effects of KRAS, BRAF, NRAS, and PIK3CA mutations on the efficacy of cetuximab plus chemotherapy in chemotherapy-refractory metastatic colorectal cancer: A retrospective consortium analysis. Lancet Oncol..

[83] Martinelli E., Morgillo F., Troiani T., Ciardiello F. (2017). Cancer resistance to therapies against the EGFR-RAS-RAF pathway: The role of MEK. Cancer Treat. Rev..

[84] Kimura Y., Inoue A., Hangai S., Saijo S., Negishi H., Nishio J., Taniguchi T. (2016). The innate immune receptor Dectin-2 mediates the phagocytosis of cancer cells by Kupffer cells for the sup-pression of liver metastasis. Proc. Natl. Acad. Sci. USA.

[85] Milette S., Sicklick J.K., Lowy A.M., Brodt P. (2017). Molecular Pathways: Targeting the Microenvironment of Liver Metastases. Clin. Cancer Res..

[86] Lingling Z., Jiewei L., Li W., Danli Y., Jie Z., Wen L., Dan P., Lei P., Qinghua Z. (2020). Molecular regulatory network of PD-1/PD-L1 in non-small cell lung cancer. Pathol. Res. Pr..

[87] Tai D., Choo S.P., Chew V. (2019). Rationale of Immunotherapy in Hepatocellular Carcinoma and Its Potential Biomarkers. Cancers.

[88] Koi M., Carethers J.M. (2017). The colorectal cancer immune microenvironment and approach to immunotherapies. Future Oncol..

[89] Kim J.H., Kim S.Y., Baek J.Y., Cha Y.J., Ahn J.B., Kim H.S., Kim T.W. (2020). A phase II study of avelumab monotherapy in patients with mismatch repair-deficient/microsatellite instabil-ity-high or POLE-mutated metastatic or unresectable colorectal cancer. Cancer Res. Treat..

[90] Zarour L.R., Anand S., Billingsley K.G., Bisson W.H., Cercek A., Clarke M.F., Coussens L.M., Gast C.E., Geltzeiler C., Hansen L. (2017). Colorectal Cancer Liver Metastasis: Evolving Paradigms and Future Directions. Cell. Mol. Gastroenterol. Hepatol..

[91] Martini G., Troiani T., Cardone C., Vitiello P.P., Sforza V., Ciardiello D., Napolitano S., Della Corte C.M., Morgillo F., Raucci A. (2017). Present and future of metastatic colorectal cancer treatment: A review of new candidate targets. World J. Gastroenterol..

[92] Ganesh K., Stadler Z.K., Cercek A., Mendelsohn R.B., Shia J., Segal N.H., Diaz L.A. (2019). Immunotherapy in colorectal cancer: Rationale, challenges and potential. Nat. Rev. Gastroenterol. Hepatol..

[93] Gol Golshani Y.Z. (2020). Advances in immunotherapy for colorectal cancer: A review. Therap. Adv. Gastroenterol..

[94] Vogel A., Hofheinz R., Kubicka S., Arnold D. (2017). Treatment decisions in metastatic colorectal cancer—Beyond first and second line combination therapies. Cancer Treat. Rev..

[95] Combo K.K., Bekaii-Saab T. (2018). A Comprehensive Review of Sequencing and Combination Strategies of Targeted Agents in Metastatic Colorectal Cancer. Oncologist.

[96] Overman M.J., Lonardi S., Wong K.Y.M., Lenz H.-J., Gelsomino F., Aglietta M., Morse M.A., Van Cutsem E., McDermott R., Hill A. (2018). Durable Clinical Benefit with Nivolumab Plus Ipilimumab in DNA Mismatch Re-pair—Deficient/Microsatellite Instability—High Metastatic Colorectal Cancer. J. Clin. Oncol..

[97] Guo J., Yu Z., Das M., Huang L. (2020). Nano Codelivery of Oxaliplatin and Folinic Acid Achieves Synergistic Chemo-Immunotherapy with 5-Fluorouracil for Colorectal Cancer and Liver Metastasis. ACS Nano.

[98] Lu Y.-C., Jia L., Zheng Z., Tran E., Robbins P.F., Rosenberg S.A. (2019). Single-Cell Transcriptome Analysis Reveals Gene Signatures Associated with T-cell Persistence Following Adoptive Cell Therapy. Cancer Immunol. Res..

[99] Lu Y.-C., Robbins P.F. (2016). Targeting neoantigens for cancer immunotherapy. Int. Immunol..

[100] Xiao L., Cen D., Gan H., Sun Y., Huang N., Xiong H., Xu X.H. (2019). Adoptive Transfer of NKG2D CAR mRNA-Engineered Natural Killer Cells in Colorectal Cancer Patients. Mol. Ther..

[101] Macrae F.A. (2016). Colorectal cancer: Epidemiology, risk factors, and protective factors. Uptodate Com.

[102] Xu T., Zhang Y., Zhang J., Qi C., Liu D., Wang Z., Li Y., Ji C., Li J., Lin X. (2020). Germline Profiling and Molecular Characterization of Early Onset Metastatic Colorectal Cancer. Front. Oncol..

[103] Preisler L., Habib A., Shapira G., Kuznitsov-Yanovsky L., Mayshar Y., Carmel-Gross I., Malcov M., Azem F., Shomron N., Kariv R. (2021). Heterozygous APC germline mutations impart predisposition to colorectal cancer. Sci. Rep..

[104] Cercek A., Kemel Y., Mandelker D., Stewart C., Arnold A.G., Sheehan M., Yaeger R., Segal N.H., Varghese A.M., Saltz L.B. (2018). Prevalence of germline genetic alterations in colorectal cancer patients. J. Clin. Oncol..

[105] Gong R., He Y., Liu X.Y., Wang H.Y., Sun L.Y., Yang X.H., Shao J.Y. (2019). Mutation spectrum of germline cancer susceptibility genes among unselected Chinese colorectal cancer pa-tients. Cancer Manag. Res..

[106] Wolf A.M.D., Fontham E.T., Church T.R., Flowers C.R., Guerra C.E., LaMonte S.J., Etzioni R., McKenna M.T., Oeffinger K.C., Shih Y.-C.T. (2018). Colorectal cancer screening for average-risk adults: 2018 guideline update from the American Cancer Society. CA A Cancer J. Clin..

[107] Lopes G., Stern M.C., Temin S., Sharara A.I., Cervantes A., Costas-Chavarri A., Engineer R., Hamashima C., Ho G.F., Huitzil F.D. (2019). Early Detection for Colorectal Cancer: ASCO Resource-Stratified Guideline. J. Glob. Oncol..

[108] Lim C.Y.S., Laidsaar-Powell R.C., Young J.M., Kao S.C., Zhang Y., Butow P. (2021). Colorectal cancer survivorship: A systematic review and thematic synthesis of qualitative research. Eur. J. Cancer Care.

[109] Pietrzyk Ł. (2017). Food properties and dietary habits in colorectal cancer prevention and development. Int. J. Food Prop..

[110] Siegel R.L., Miller K.D., Sauer A.G., Fedewa S.A., Butterly L.F., Anderson J.C., Cercek A., Smith R.A., Jemal A. (2020). Colorectal cancer statistics. CA A Cancer J. Clin..

[111] Klepp P., Brackmann S., Cvancarova M., Hoivik M.L., Hovde M., Henriksen M., Huppertz-Hauss G., Bernklev T., Hoie O., Kempski-Monstad I. (2020). Risk of colorectal cancer in a population-based study 20 years after diagnosis of ulcerative colitis: Results from the IBSEN study. BMJ Open Gastroenterol..

[112] McDowell C., Farooq U., Haseeb M. (2021). Inflammatory Bowel Disease.

[113] Singh R.K., Chang H.-W., Yan D., Lee K.M., Ucmak D., Wong K., Abrouk M., Farahnik B., Nakamura M., Zhu T.H. (2017). Influence of diet on the gut microbiome and implications for human health. J. Transl. Med..

[114] Lazar V., Ditu L.-M., Pircalabioru G.G., Gheorghe I., Curutiu C., Holban A.M., Picu A., Petcu L., Chifiriuc M.C. (2018). Aspects of Gut Microbiota and Immune System Interactions in Infectious Diseases, Immunopathology, and Cancer. Front. Immunol..

[115] DeGruttola A.K., Low D., Mizoguchi A., Mizoguchi E. (2016). Current Understanding of Dysbiosis in Disease in Human and Animal Models. Inflamm. Bowel Dis..

[116] Rowland I., Gibson G., Heinken A., Scott K., Swann J., Thiele I., Tuohy K. (2017). Gut microbiota functions: Metabolism of nutrients and other food components. Eur. J. Nutr..

[117] Fukui H., Xu X., Miwa H. (2018). Role of Gut Microbiota-Gut Hormone Axis in the Pathophysiology of Functional Gastrointestinal Disorders. J. Neurogastroenterol. Motil..

[118] Cheng Y., Ling Z., Li L. (2020). The Intestinal Microbiota and Colorectal Cancer. Front. Immunol..

[119] Wang Y., Qin S., Jia J., Huang L., Li F., Jin F., Wang Y. (2019). Intestinal Microbiota-Associated Metabolites: Crucial Factors in the Effectiveness of Herbal Medicines and Diet Therapies. Front. Physiol..

[120] Ghosh S.S., Wang J., Yannie P.J., Ghosh S. (2020). Intestinal barrier function and metabolic/liver diseases. Liver Res..

[121] Vancamelbeke M., Vermeire S. (2017). The intestinal barrier: A fundamental role in health and disease. Expert Rev. Gastroenterol. Hepatol..

[122] Caballero S., Pamer E.G. (2015). Microbiota-Mediated Inflammation and Antimicrobial Defense in the Intestine. Annu. Rev. Immunol..

[123] Pandiyan P., Bhaskaran N., Zou M., Schneider E., Jayaraman S., Huehn J. (2019). Microbiome Dependent Regulation of Tregs and Th17 Cells in Mucosa. Front. Immunol..

[124] Ciardiello D., Vitiello P.P., Cardone C., Martini G., Troiani T., Martinelli E., Ciardiello F. (2019). Immunotherapy of colorectal cancer: Challenges for therapeutic efficacy. Cancer Treat. Rev..

[125] Pitt J.M., Marabelle A., Eggermont A., Soria J.C., Kroemer G., Zitvogel L. (2016). Targeting the tumor microenvironment: Removing obstruction to anticancer immune responses and immu-notherapy. Ann. Oncol..

[126] Sarvizadeh M., Ghasemi F., Tavakoli F., Khatami S.S., Razi E., Sharifi H., Biouki N.M., Taghizadeh M. (2018). Vaccines for colorectal cancer: An update. J. Cell. Biochem..

